# The metalloprotease PrtV from *Vibrio cholerae* Purification and properties

**DOI:** 10.1111/j.1742-4658.2008.06470.x

**Published:** 2008-06

**Authors:** Karolis Vaitkevicius, Pramod K Rompikuntal, Barbro Lindmark, Rimas Vaitkevicius, Tianyan Song, Sun N Wai

**Affiliations:** Department of Molecular Biology, Umeå UniversitySweden

**Keywords:** characterization, metalloprotease, PrtV, purification, *V. cholerae*

## Abstract

The *Vibrio* metalloprotease PrtV was purified from the culture supernatant of a *Vibrio cholerae* derivative that is deficient in several other secreted peptidases, including the otherwise abundant hemagglutinin/protease HapA. The PrtV is synthesized as a 102 kDa protein, but undergoes several N- and C-terminal processing steps during *V. cholerae* envelope translocation and prolonged incubation. Purified *V. cholerae* PrtV protease forms of 81 or 73 kDa were stabilized by calcium ions. Removal of calcium resulted in further rapid autoproteolysis. The two major products of autoproteolysis of the PrtV protease were approximately 37 and 18 kDa and could not be separated under non-denaturing conditions, indicating they are interacting domains. In an assay using cultured cells of the human intestinal cell line HCT8, the PrtV protein showed a cytotoxic effect leading to cell death. Using human blood plasma as a source of potential substrates of mammalian origin for the PrtV protease, we found that the extracellular matrix components fibronectin and fibrinogen were degraded by the enzyme. Additional tests with individual protein substrates revealed that plasminogen was also a possible target for the PrtV protease.

*Vibrio cholerae*, the causative agent of cholera, is a Gram-negative motile bacterial species acquired via ingestion of contaminated food or water. Little is known regarding the environmental survival factors that exist in *V. cholerae* and play an important role in maximizing the ability of the vibrios to survive and multiply in the environment interacting with predators. In a recent study, we established that *Caenorhabditis elegans* is useful as a model system for identifying and assessing factors from *V. cholerae* other than cholera toxin that may contribute to pathogenesis and damage to host organisms [1]. Using reverse molecular genetics techniques, we identified an extracellular protease, the previously uncharacterized PrtV protein, as being necessary for killing of nematodes by *V. cholerae* [1]. The killing effect is associated with colonization of the *C. elegans* intestine. The elucidation of mechanisms behind this role for PrtV is of importance for the further understanding of *V. cholerae* environmental survival and bacteria–host interaction.

Tissue barriers to pathogen invasion, such as extracellular matrices, epidermal keratinocyte layers and blood vessel walls, may be targeted by bacterial proteases. Proteolysis of host tissue components, such as extracellular matrix proteins, including collagen, laminin, fibronectin and elastin, could induce necrotic tissue damage [2,3]. *Pseudomonas aeruginosa* and *Serratia mercescens* proteases can degrade corneal proteoglycan ground substance and cause keratitis [4,5]. The blood clotting system plays a role in immobilization of invading pathogens and prevention of their dissemination. A pathogen can either use an arsenal of its own proteases, or induce the host fibrinolytic system(s) to dissolve the fibrin clot [6]. *Yersinia pestis*, the causative agent of plague, produces a plasminogen activator Pla, whose inactivation decreases the infectivity of the bacterium a million-fold in a mouse infection model in which the pathogen is injected subcutaneously, mimicking a flea bite [7]. In some cases, bacterial proteases act directly as toxins responsible for the disease. Clostridial neurotoxins are zinc metalloproteases that specifically cleave a membrane protein of synaptic vesicles, called synaptobrevin. Synaptobrevin proteolysis prevents neurotransmitter release, which results in paralysis of the host [8].

Pathogen-produced proteases may modulate bacterial virulence factor activities, for example hemagglutinin/protease (as well as other proteases, including those of the host) is able to process and activate cholera toxin and hemolysin from *V. cholerae* [9,10].

The PrtV protease from *V. cholerae* belongs to the evolutionary peptidase clan M6, and shares 37% identity with immune inhibitor A (InhA) from the Gram-positive bacterium *Bacillus thuringiensis* [11]. InhA was identified as one of extracellular bacterial factors that can block the humoral defence system of pupae of the silk moth *Hyalophora cecropia* against *Escherichia coli*, but not against *Bacillus subtilis* or *Bacillus cereus* [12,13]. Injection of purified InhA into *Callosamiapromethea* pupae and *Drosophila melanogaster* caused lethality [13]. The purified InhA preparation showed weak proteolytic activity on casein, but was more active in degrading antimicrobial peptides from the hemolymph of *Hyalophora cecropia in vitro* [14]. A study of the specificity of the enzyme towards a synthetic and a natural cecropin antimicrobial peptide did not identify any distinct pattern, as a number of peptide bonds were cleaved [14] (http://merops.sanger.ac.uk/). It was therefore concluded that the proteolytic attack may be of broad specificity because cecropins occur largely in a random coil conformation in solution, and this open structure made them susceptible to the InhA protease [15]. As degradation of inducible antimicrobial peptides is not likely to cause lethality to insects, other unidentified targets of InhA were suggested [15]. In addition, InhA1, a homologue of InhA, was shown to be required for lethal oral *B. thuringiensis* infection of insect larvae [16,17]. The M6 evolutionary protein family has been found to be distributed in various species of environmental bacteria including *Vibrio*, *Shewanella*, *Clostridium*, *Geobacillus* and *Bacillus*, suggesting that there might be a role for this type of protease in bacterial environmental persistance and survival [18] (http://merops.sanger.ac.uk/).

The *V. cholerae* PrtV protein is postulated to share certain properties with the well-studied proteases from *Bacillus* species, such as the presence of a Zn^2+^-binding site and modulation of activity/stability by divalent metal ions. However, the actual substrate specificity of the PrtV protein from *V. cholerae* has not been elucidated, and no purification and characterization were performed for this M6 peptidase from *Vibrio* or any other Gram-negative bacterial species. A similarly named protease (designated PrtVp or PrtV) has been described and purified from *Vibrio parahemolyticus* [19–21]. However, the PrtVp enzyme is a collagenase, lacks homology to PrtV of *V. cholerae*, and belongs to a separate metalloprotease evolutionary family, M9 (http://merops.sanger.ac.uk/). In addition, there are two putative M6 family protease encoding genes in the published genome sequence of *V. parahemolyticus* RIMD2210633 (the genetic loci VP0907 and VPA0715 are both separate from the PrtVp locus), but none of their gene products have yet been characterized (http://www.ncbi.nlm.nih.gov/sites/entrez).

In this study, the PrtV protein of *V. cholerae* O1 strain C6706 was purified and assayed for its proteolytic activities using azocasein, gelatin and human plasma proteins as substrates, and tested in a cytotoxicity assay using human ileocaecum carcinoma cells (HCT8 cell line).

## Results and Discussion

### Purification and physicochemical characterization of the PrtV protein from *V. cholerae*

**Fig. 1:**
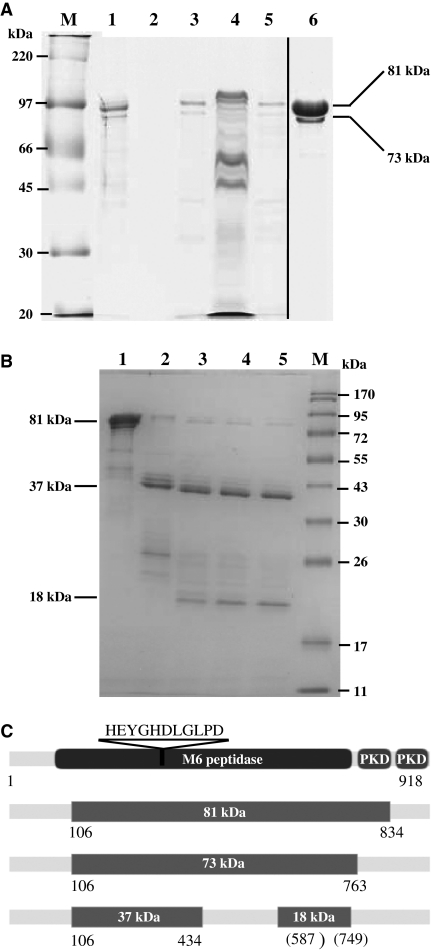
*V. cholerae* PrtV purification steps, calcium requirement for stability, and autoprocessing. (A) PrtV purification steps. Samples taken at various stages during the protein purification were analysed by SDS–PAGE and Coomassie blue staining. Lane 1, culture supernatant proteins concentrated 40-fold by trichloroacetic acid precipitation; lane 2, permeate after ultrafiltration and 40-fold concentration of proteins by trichloroacetic acid precipitation; lane 3, retentate after ultrafiltration; lane 4, pellet after retentate centrifugation; lane 5, supernatant after retentate centrifugation; lane 6, PrtV preparation after chromatography on a DEAE-Toyopearl 650S column; M, prestained molecular mass marker, molecular masses indicated in kDa. (B) Calcium requirement for PrtV protease stability. The PrtV fraction consisting mainly of the 81 kDa form of the protease (amino acids 106–834) was desalted on a Sephadex G25 HiPrep 26/10 column (GE Healthcare, Chalfont St Giles, UK) equilibrated with a buffering solution containing 10 mm MgCl_2_. Exchange of calcium with magnesium led to destabilization of the PrtV metalloprotease and autoproteolysis, resulting in stable PrtV peptide chains marked 37 kDa (amino acids 106–434) and 18 kDa (amino acids 587–749). The estimated molecular masses of PrtV fragments were calculated from mass spectrometry data of the tryptic digestion products combined with the N- and C-terminal sequencing data (see Results). Lane 1, PrtV fraction after chromatography on a DEAE-Toyopearl 650S column containing 10 mm CaCl_2_; lane 2, PrtV fraction for which Ca^2+^ has been exchanged with Mg^2+^ as described above and incubated for 3 h on ice; lane 3, PrtV fraction for which Ca^2+^ has been exchanged with Mg^2+^and incubated for 1 h at 30 °C; lane 4, PrtV fraction for which Ca^2+^ has been exchanged with Mg^2+^and incubated for 2 h at 30 °C; lane 5, PrtV fraction for which Ca^2+^ has been exchanged with Mg^2+^and incubated for 3 h at 30 °C; M, prestained molecular mass markers indicated in kDa. (C) Schematic representation of PrtV protein forms identified in this study. The M6 peptidase domain, its predicted catalytic Zn^2+^-binding site and PKD domains are indicated with respect to the 918 amino acid full-length PrtV protein. The lower diagrams show the 81, 73, 37 and 18 kDa processed forms of the PrtV protease with terminal amino acid residues indicated by numbers (referring to positions in the full-length PrtV protein). In the case of the 18 kDa polypeptide, the numbers are shown in parentheses as they represent tentative termini deduced from mass spectrometry analysis.

**Fig. 2:**
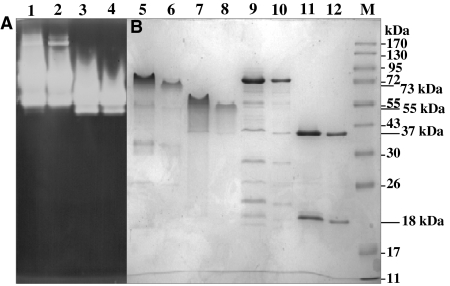
Interaction between the two main PrtV autoproteolysis products. Samples in lanes 1-4 were analyzed by zymography on an SDS–PAGE gel containing gelatin as a PrtV substrate. SDS–PAGE was performed for both unboiled and boiled PrtV samples (lanes 1-8 and 9-12, respectively). Lanes 3, 4, 7, 8, 11 and 12 were loaded with PrtV protease destabilized by exchange of soluble calcium with magnesium, which resulted in 37 and 18 kDa protein fragments (as determined by mass spectrometry of tryptic digestion products, and N- and C-terminal peptide sequencing) further co-purified on a HiLoad 16/60 Superdex 75 pg chromatography column (GE Healthcare). Lanes 1, 2, 5, 6, 9 and 10 were loaded with the PrtV protein fraction consisting of mostly the 73 kDa protein fragment (amino acids 106–763). The protein amount in lanes 1, 3, 5, 7, 9 and 11 is 15 pmol and that in lanes 2, 4, 6, 8, 10 and 12 is 5 pmol. The lane marked M contains a prestained molecular mass marker (molecular masses indicated in kDa). Proteins in gels were stained using Coomassie blue.

PrtV is encoded by a 102 kDa ORF [11], is secreted from *V. cholerae* cells, and undergoes several N- and C-terminal (auto)proteolytic cleavage steps resulting in active protease forms of 81 and 73 kDa (lane 6 in Fig. 1A). Optimized expression of PrtV was induced using 0.002% arabinose, and purification was performed as described in Experimental procedures. This protein preparation resulted in enrichment of the 81 and 73 kDa PrtV protease forms (Fig. 1). The 15 L of culture supernatant yielded approximately 50 mg of total PrtV protein preparation, with > 90% being the 81 kDa protein form. Mass spectrometric analysis of the 81 and 73 kDa derivatives of the PrtV protein verified the presence of amino acids 137–834 and 137–763 of the full-length protein, respectively. Further N-terminal sequence analysis showed that these protein forms start with amino acid 106 (numbers refer to positions in the full-length PrtV protein). The 81 kDa protein contained the intact sequence of one of two polycystic kidney disease (PKD) domains at the C-terminus (Fig. 1C) [18,22] (http://merops.sanger.ac.uk/; http://pfam.sanger.ac.uk/), but neither of these domains were present in the 73 kDa form of PrtV. The function of the PKD domain is not known; it may be involved in protein–protein or protein–carbohydrate interactions and is found in some bacterial proteases and chitinases, as well as archebacterial and vertebrate proteins [22] (http://pfam.sanger.ac.uk/). These major PrtV fragments were partially separated by ion-exchange chromatography; however, further degradation of both enzyme forms occurred, resulting in the gradual accumulation of lower-molecular-mass autoproteolysis products (lanes 9 and 10 in Fig. 2).

### Optimum conditions for the stability and processing of the PrtV protein

The enzyme was relatively stable at low temperature in the presence of Ca^2+^ ions; however, it could be further degraded, eventually resulting in two interacting polypeptide chains of 37 and 18 kDa (Figs 1B and 2). A similar conversion has been described for immune inhibitor A from *Bacillus anthracis* and *B. cereus* [23,24]. Mass-spectrometric analysis of tryptic digestion products showed that the 37 kDa N-terminal part of the PrtV protease contains amino acids 137–425, which include the predicted catalytic Zn^2+^ binding site. N- and C-terminal sequencing confirmed these findings, and the precise terminal amino acids of the 37 kDa PrtV fragment were found to be 106 and 434 (numbers refer to positions in the full-length PrtV protein). The 18 kDa C-terminal part contains amino acids 587–749 of the full-length PrtV protein (Fig. 1C) as determined by mass spectrometry of the tryptic digest products; N- and C-terminal sequencing attempts on the 18 kDa form were unsuccessful. The molecular masses of the PrtV protease fragments were calculated assuming the length of these proteins to be as detected by mass spectrometry of tryptic digestion products and combined with the data from N- and C-terminal sequencing. Separation of the 37 and 18 kDa PrtV polypeptides using size-exclusion, anion-exchange and hydrophobic-interaction chromatography were unsuccessful (data not shown). The resulting protein migrated as a band of approximately 55 kDa by SDS–PAGE if it had not been heat-denatured (compare lanes 3, 4, 7 and 8 with lanes 11 and 12 in Fig. 2). The proteolytic activity for tested substrates of the 55 kDa form of PrtV was identical to that of the 81 or 73 kDa forms (data not shown and Fig. 2). However, the 81 kDa form was gradually converted to the 73 kDa PrtV form during incubation with substrates even in the presence of 10 mm Ca^2+^ (data not shown). The molar activity ratio of 55 and 73 kDa PrtV forms was estimated to be 1.12 ± 0.07 using a fluorescein-labelled gelatin substrate, and therefore there is probably no difference in the specific activity of these two protease forms. Therefore, the 55 kDa PrtV form (37 + 18 kDa) that remains stable in the absence of Ca^2+^ was used for further PrtV characterization.

**Fig. 3:**
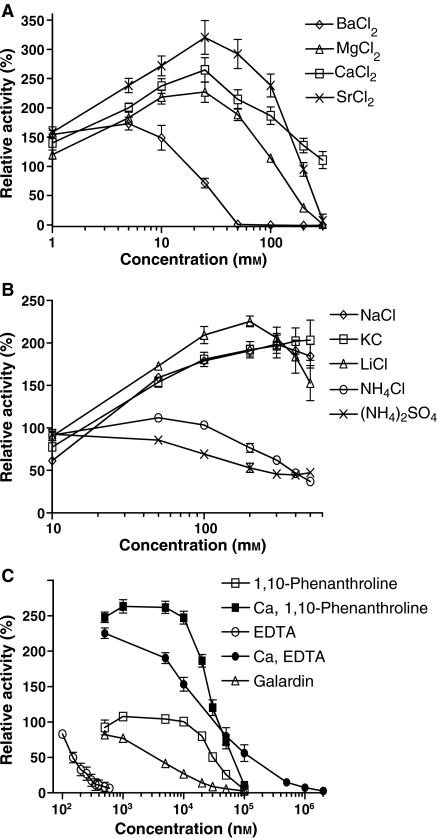
(A)–(C) Dependence of PrtV proteolytic activity (37 + 18 kDa form) on metal ion, salt and inhibitor concentration. PrtV activity without addition of salts or inhibitors is set as 100%. See Results for detailed information.

The PrtV protein was stabilized by the addition of Ca^2+^, although excess addition of this ion was not essential for enzyme activity (Figs 1B and 3). PrtV activity was enhanced by Ca^2+^, Sr^2+^, Mg^2+^ and to some extent Ba^2+^; however, Ba^2+^ inhibited PrtV at higher concentrations whereas the other divalent metal ions tested showed an enhancement of PrtV proteolytic activity (Fig. 3A). The optimal concentration of Ca^2+^, Sr^2+^ and Mg^2+^ was approximately 25 mm, and the highest activity of PrtV occurred in the presence of Sr^2+^ (Fig. 3A). An enhancement of proteolytic activity was observed by the addition of monovalent metal ion Na^+^, K^+^ and Li^+^ salts (Fig. 3B), as has also been demonstrated for some other metalloenzymes, e.g. a collagenase from *Clostridium histolyticum* [25], thermolysin [26], and a metallopeptidase from *Lactobacillus helveticus* [27]. The optimal LiCl salt concentration for PrtV proteolytic activity was 0.2 m, but the activity was enhanced in the presence of NaCl or KCl at concentrations of up to 0.5 m (Fig. 3B).

No activity of PrtV was detected using the synthetic neutral proteinase substrate FAGLA and collagenase substrate FALGPA (data not shown).

### Inhibition of PrtV activity

The metal ion chelators EDTA and 1,10-phenanthroline completely inhibited the PrtV protease, and the activity could not be restored by addition of excess calcium ions (Fig. 3C). Addition of excess calcium increased the concentration of EDTA required for inhibition of PrtV protease activity, but 1,10-phenanthroline, which does not chelate calcium ions, was equally efficient at inhibiting the protease with or without Ca^2+^. A metalloprotease inhibitor galardin [28] inhibited the PrtV protease at a 200–20 000-fold molar excess (Fig. 3C).

### Cytotoxic activity of PrtV

**Fig. 4:**
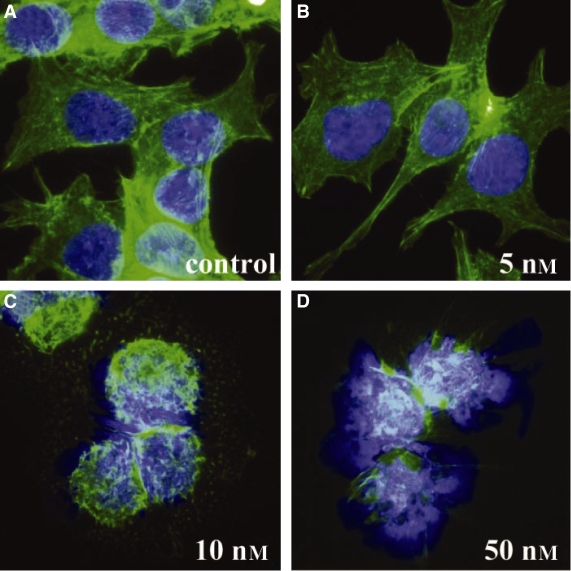
Cytotoxic effect of purified PrtV protein of *V. cholerae* on HCT8 cells. (A) Control without PrtV protein; (B)–(D) HCT8 cells treated with 5 nm (B), 10 nm (C) or 50 nm (D) PrtV protein. After 6 h treatment, actin filaments and nuclei were stained with phalloidin and DAPI, respectively, as described in Experimental procedures.

To test possible toxic activities of PrtV towards mammalian tissue, purified enzyme was added to cultured human colon cancer HCT8 cells. The cytotoxic effect of the PrtV protein, as observed by fluorescence microscopy, occurred within 6 h of incubation of the HCT8 cells with 0, 5, 10 and 50 nm of purified PrtV. As shown in Fig. 4, the degree of cytotoxicity was increased by the amount of PrtV added to the cells, indicating that cytotoxicity of PrtV was dose-dependent. This result suggests that the PrtV protein of *V. cholerae*, similar to other proteases [2] including hemagglutinin/protease from *V. cholerae* [29], might cause tissue damage by directly degrading substrate proteins in host tissues, thereby inducing cell rounding and detachment.

### Substrate specificities of PrtV as monitored with human plasma proteins

**Fig. 5:**
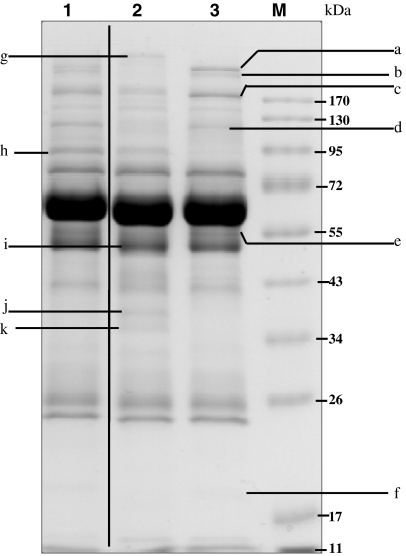
Human blood plasma proteins as candidate substrates for the PrtV protease. The letters ‘a’–‘k’ indicate protein bands excised from the SDS–PAGE gel for protein identification by mass spectrometric analysis. Lanes 1 and 2 were loaded with blood plasma proteins incubated with PrtV for 5 and 120 min respectively, while lane 3 contained blood plasma proteins incubated for 120 min without the protease under the same conditions. M, prestained molecular mass marker (molecular masses indicated in kDa).

**Fig. 6:**
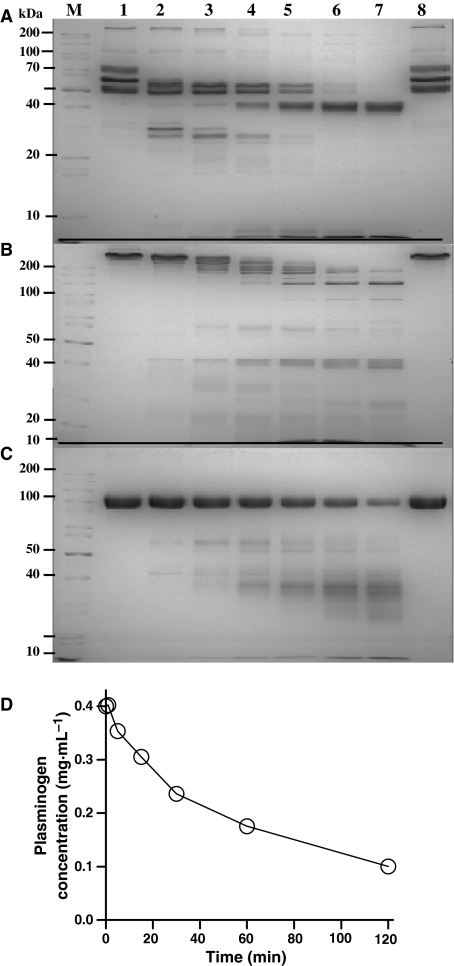
Proteolysis of fibrinogen (A), fibronectin (B) and plasminogen (C) by PrtV. Lane M, molecular mass markers indicated in kDa; lanes 1 and 8, pure candidate substrate protein without PrtV; lanes 2–7, candidate substrate protein incubated with PrtV protease as described in Experimental procedures. The time points for samples taken during incubation at 37 °C were: lane 1, 0 min; lane 2, 1 min; lane 3, 5 min; lane 4, 15 min; lane 5, 30 min; lane 6, 60 min; lanes 7 and 8, 120 min. The quantification of plasminogen remaining intact at various time points is shown in (D).

**Fig. 7:**
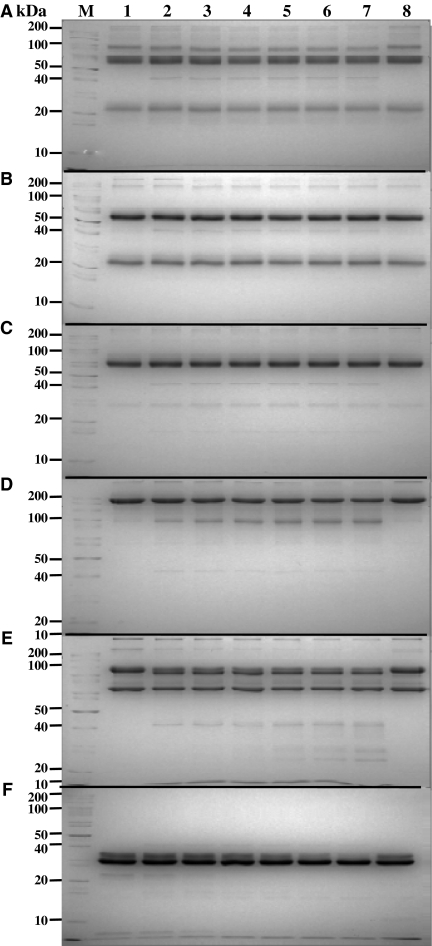
Testing of IgA (A), IgG (B), urokinase-type plasminogen activator (C), α-2-macroglobulin (D), complement component C3 (E) and thrombin (F) for PrtV proteolysis. Lane M, molecular mass markers indicated in kDa; lanes 1 and 8, pure candidate substrate protein without PrtV; lanes 2–7, candidate substrate protein incubated with PrtV protease as described in Experimental procedures. The time points for samples taken during incubation at 37 °C were: lane 1, 0 min; lane 2, 1 min; lane 3, 5 min; lane 4, 15 min; lane 5, 30 min; lane 6, 60 min; lanes 7 and 8, 120 min.

To study potential targets of the PrtV protein, we initially used human serum as a source of substrate proteins to define the specificity of the enzyme. Candidate substrates for this enzyme were determined by mass spectrometry after comparing plasma protein patterns using SDS–PAGE following incubation with and without PrtV (Fig. 5). The blood plasma components analyzed using this method were as follows: (a) lipoprotein/apolipoprotein, (b) fibronectin-1, (c) α-2-macroglobulin, (d) complement component C3, (e) fibrin β chain, (f) not identified, (g) α-2-macroglobulin, (h) α-2-macroglobulin mixture with PrtV, (i) α-1-antitrypsin, (j) complement component C3 and fibrinogen, and (k) fibrinogen (Fig. 5). Potential candidates and several other human plasma proteins were selected for confirmation of direct cleavage and degradation by the PrtV protease using pure candidate substrates. Fibrinogen (all α, β and γ chains), fibronectin and plasminogen were effectively degraded (Fig. 6). Immunoglobulin A, immunoglobulin G, urokinase-type plasminogen activator and thrombin were not affected by incubation with PrtV (Fig. 7A–C,F). α-2-macroglobulin most likely underwent an activation step [30] induced by the protease, resulting in the appearance of a 85 kDa band on an SDS–PAGE gel after incubation with the PrtV protease (Fig. 7D). Limited cleavage of the C3 complement component probably occurred in the α chain (Fig. 7E). Taken together, the results from the human blood plasma experiments and confirmation of proteolytic cleavage with purified candidate substrates show that fibrinogen, fibronectin and plasminogen are among the preferred substrates of PrtV.

Plasminogen, the zymogen of the fibrinolytic system, is converted into the active enzyme plasmin by plasminogen activators [31]. Plasmin(ogen) not only plays an essential role in the clearance of fibrin from the circulation, but also is involved in several other biological phenomena, including macrophage recruitment, arterial stenosis, atherosclerosis, aneurysm formation, skin and corneal wound healing, glomerulonephritis and neovascularization [32–35]. SDS–PAGE analysis of the human plasminogen/PrtV protein reaction mixtures revealed that the majority of native plasminogen was converted to lower-molecular-mass fragments. To study the interaction of PrtV protease with plasminogen in more detail, the time course of proteolysis was analyzed at various time points. As shown in Fig. 6D, 50% degradation of plasminogen was observed within 60 min at 0.4 mg·mL^−1^ substrate and 100 nm PrtV concentrations, whereas about 80% digestion was achieved after 120 min. Further characterization of the generated plasminogen fragments by purification and functional characterization is necessary to explain the mechanism of PrtV protein proteolytic activity on human plasminogen. Taken together, we suggest that the PrtV protease of *V. cholerae* might play a role in *V. cholerae* pathogenesis by modulation of the stability of the extracellular matrix in host tissues.

The range of primary sequences that constitute actual cleavage sites for the PrtV enzyme remains to be determined. Examination of the PrtV amino acid sequences representing cleavage positions in PrtV autoproteolysis, as determined by N- and C-terminal sequencing, provided initial information to be explored in future studies. For example, the ends of the 81, 73 and 37 kDa polypeptides reveal that cleavage may occur at the following amino acid sequences: RVF/ALD, RVE/GIK and TIK/VDT. Such sequences were indeed found to be present in human complement components and immunoglobulins according to our BLAST database searches (data not shown), but further studies are required to determine which flanking amino acids are parts of cleavage sites. The present studies and purification of PrtV should aid further biochemical analyses.

We hypothesize that, in the case of *V. cholerae,* this PrtV protease and its gene, located in a putative pathogenicity island in the second chromosome, somehow contribute to bacterial survival in natural environments, i.e. in contact with natural host organisms, such as protozoa, copepods and crustaceans. The substrates of the protease *in vivo* remain unknown. Given the activity of the protease on human extracellular matrix proteins and blood components, the present findings suggest that PrtV could interfere with normal extracellular matrix composition, hemostasis and innate immune responses in intestinal or wound infections.

## Experimental procedures

### Purification of the PrtV protein

The secreted PrtV metalloprotease was purified from 15 L of culture supernatant from the *V. cholerae* strain KAS202 carrying plasmid pKVA232 harboring a full-length *prtV* gene under the control of an arabinose-inducible promoter [1]. TG culture medium [36] supplemented with 5 mm CaCl_2_, 0.5% w/v casamino acids (Becton Dickinson, Franklin Lakes, NJ, USA), 50 μg·mL^−1^l-tryptophan and 50 μg·mL^−1^ carbenicillin was used for bacterial growth. Protein expression was induced by addition of 0.002% w/v l-arabinose when the cell density reached an attenuance (*D*_600_) of 0.5. After 14 h incubation at 30 °C on a rotary shaker at 100 rpm in Ehrlenmeyer flasks, the bacterial cells were removed by centrifugation at 5000 ***g*** for 30 min. Subsequently, the resulting culture supernatants were clarified by depth filtration through LP50- and LP90-grade Zeta Plus filtration media (CUNO, Meriden, CT, USA). The filtrates were concentrated 30-fold, and the solute exchanged with a buffering solution compatible with a further purification step using a Prep/Scale 0.23 m^2^ 30 kDa cut-off polysulfone membrane cartridge (Millipore, Billerica, MA, USA). The retentate after ultrafiltration was centrifuged for 30 min at 20 000 ***g***, and the supernatants were filtered through a 0.22 μm pore diameter polysulfone membrane (Millipore) and applied onto a 65 mL Toyopearl DEAE 650S column (Tosoh, Tokyo, Japan). The PrtV protease was eluted using a linear NaCl gradient from 0 to 180 mm. All steps were carried out in a buffer solution containing 10 mm CaCl_2_ to stabilize the enzyme, 20 mm triethanolamine pH 7.80 as a buffering agent, and 1-5 mm dithiothreitol as an antioxidant. Fractions containing PrtV were desalted on a 53 mL Sephadex G25 column (GE Healthcare) and frozen in a dry ice/ethanol bath. Selected fractions containing PrtV protein were further purified by chromatography using a 1 mL MonoQ column (GE Healthcare) by gradient elution with NaCl. The protein concentration was determined by light absorbance at 280 nm.

### Proteolytic activity determination

A modified azocasein assay was used for routine activity measurements as described previously [1]. A more sensitive and linear method using fluorescein-labeled gelatin (DQ Gelatin; Molecular Probes, Eugene, OR, USA) was used for confirmation of data obtained with the azocasein substrate (Sigma, St Louis, MO, USA). DQ Gelatin (12.5 μg·mL^−1^) was incubated at 37 °C with 5 nm PrtV protease in 50 mm Hepes pH 7.20 unless specified otherwise. The reaction rate was measured at 495 nm excitation and 535 emission wavelengths using 96-well black non-binding surface microplates (Corning, NY, USA). Measurements were performed on an Infinite M200 fluorescence microplate reader (Tecan, Mannedorf, Switzerland). The activity of the protease was tested on synthetic peptide substrates FAGLA (3-(2-furylacryloyl)-glycyl-l-leucine amide) and FALGPA (2-furylacryloyl-l-leucylglycyl-l-prolyl-l-alanine) using previously described procedures [35,37].

Where applicable, reagent solutions were dithizone-extracted [38] for adventitious metal ion control.

### Zymography

Detection of proteolytic activity was performed by SDS–PAGE using 0.25% w/v gelatin as a substrate in the separating gel. Protein samples in SDS–PAGE sample buffer were incubated for 5 min at 37 °C before loading. After electrophoresis, SDS was washed out from the gel by soaking for 30 min in 20% 2-propanol [39], 50 mm Tris/Cl pH 7.50 and 10 mm CaCl_2_, exchanging the solution every 10 min. Renaturation of proteins in the gel was performed by incubation in 50 mm Tris/Cl pH 7.50, 10 mm CaCl_2_ and 5% glycerol at 4 °C for 4 h. Proteolysis was allowed to occur for 1–24 h at 37 °C in the same solution, although PrtV activity was readily detected after incubation at 4 °C. After Coomassie blue staining, areas of protease activity in the gel appeared as clear bands where the protease had digested the gelatin, against a dark blue background.

### Analysis of substrate proteins in blood plasma by the PrtV protease

Human blood plasma was used to analyze potential substrate proteins of the PrtV protein of *V. cholerae.* Blood plasma was diluted 100-fold in 50 mm Tris/Cl pH 7.50 and 10 mm MgCl_2_. Subsequently, 10 μg·mL^−1^ (129 nm) of the 81 kDa PrtV protease was added and the mixture was incubated at 37 °C. At various time intervals, a portion of the reaction solution was removed and analysed by SDS–PAGE and Coomassie blue staining. The degraded proteins bands were excised from the gel and identified by mass spectrometric analysis of tryptic digestion products.

### Substrate specificity assay

Tests of substrate specificity of the PrtV protein were performed using commercially available purified human α-2-macroglobulin, complement component C3, immunoglobulin A, immunoglobulin G, fibrinogen, fibronectin, plasminogen, urokinase-type plasminogen activator and thrombin. Candidate substrate proteins of the PrtV protease at a concentration of 0.4 mg·mL^−1^ in 50 mm HEPES pH 7.20, 1 mm CaCl_2_ and 1 mm MgCl_2_ were assayed for cleavage by 100 nm PrtV protein (37 + 18 kDa form). The reaction was analysed by SDS–PAGE and Coomassie blue staining at several time points during incubation at 37 °C.

### SDS–PAGE analysis

SDS–PAGE analysis was performed as described previously [40]. When necessary, quantification of Coomassie-stained protein bands in gel was performed using quantity one software (Bio-Rad, Hercules, CA, USA).

### Protein identification

Mass spectrometry, protein identification and database searches were carried out by the Wallenburg Consortium north Expression Proteomics Facility (Department of Medical Biochemistry and Microbiology, Uppsala University, Sweden). For the mass spectrometry analysis, proteins in gel slices were alkylated with iodoacetamide and digested using modified trypsin (Promega, Fitchburg, WI, USA). The digests were analysed by MALDI-TOF mass spectroscopy using an Ultraflex Tof/Tof instrument (Bruker Daltonics, Bremen, Germany). The instrument was calibrated using peptide mixture II (Bruker Daltonics), and each spectrum was calibrated using autodigestion products of the trypsin. After removal of background peaks and correction of software errors in detecting monoisotopic *m*/*z*, the peak lists were used to search the National Center for Biotechnology Information non-redundant database using the Mascot search engine (http://www.matrixscience.com). For estimation of cleavage positions in the degraded forms of the metalloprotease, the *m*/*z* of tryptic peptides was fitted to the amino acid sequence of the protease. N- and C-terminal amino acid sequence analyses by Edman degradation and C-terminal chemical degradation of the protein bands identified as the 81, 73 and 55 kDa PrtV forms in SDS–PAGE (see Results) were performed by the Protein Analysis Center at the Karolinska Institute, Stockholm, Sweden (http://www.mbb.ki.se/pac/index.html).

### Protease inhibition analyses

The effects of the metalloprotease inhibitor galardin (5 × 10^2^ to 10^5^ nm) and metal-chelating compounds EDTA (10^2^ to 2 × 10^6^ nm) and 1,10-phenanthroline (5 × 10^2^ to 10^5^ nm) were examined by preincubation of the purified PrtV protein (37 + 18 kDa form) at 5 nm concentration for 30 min with one of inhibitors in 50 mm HEPES pH 7.20, followed by addition of the DQ Gelatin (Molecular Probes) substrate and fluorescence measurement using an Infinite M200 instrument (Tecan). The PrtV activity with EDTA and 1,10-phenanthroline was measured both in the presence and absence of 10 mm CaCl_2_.

### Cell line, media and culture conditions

The human ileocecum carcinoma cell line HCT8 (ATCC number CCL-224) was kindly provided by the Institute for Molecular Infection Biology, University of Würzburg, Germany.

HCT8 cells were cultured in RPMI-1640 (Invitrogen, Carlsbad, CA, USA) supplemented with 2 mm glutamine, 1 mm pyruvate, 10% fetal bovine serum and 50 μg·mL^−1^ gentamicin. The cells were cultivated at 37 °C in a 5% CO_2_ atmosphere.

### Tissue toxicity assay

HCT8 cells were seeded in 24-well plates (Becton Dickinson) and grown to 50% confluence. Purified PrtV protein (5–50 nm) was added to the cells. The occurrence of cytotoxic effects was monitored and compared with the responses of control cells for up to 6 h. Cells were fixed with 2% paraformaldehyde in NaCl/P_i_ (pH 7.3) for 10 min. After fixation, cells were washed twice with NaCl/P_i_ and incubated with 0.1 m glycine for 5 min at room temperature. After washing twice with NaCl/P_i_, the cells were permeabilized with 0.5% Triton X-100 (Sigma-Aldrich). Actin was stained using Alexa Fluor 488 phalloidin (Molecular Probes) containing 1% BSA (Sigma-Aldrich). After thorough washing with NaCl/P_i_, the nuclei were stained with DAPI (Sigma-Aldrich) (1 : 5000) for 5 min before mounting in Mowiol (Scharlau Chemie SA, Barcelona, Spain) that contains antifade (P-phenylene diamine). Cells were analysed using a Zeiss Axioskop (Zeiss, Oberköchen, Germany) microscope, and photographed using a Hamamatsu digital camera (Hamamatsu, Hamamatsu City, Japan).

## Abbreviations

DAPI: 4′,6-diamidino-2-phenylindole
PKD: polycystic kidney disease
